# Mitochondrial dynamism and heart disease: changing shape and shaping change

**DOI:** 10.15252/emmm.201404575

**Published:** 2015-04-10

**Authors:** Gerald W Dorn II

**Affiliations:** 1 Center for Pharmacogenomics, Department of Internal Medicine, Washington University School of MedicineSt. Louis, MO, USA

**Keywords:** mitochondrial fusion, mitochondrial fission, mitophagy, cardiomyopathy, Parkinson's disease

## Abstract

Mitochondria of adult cardiomyocytes appear hypo-dynamic, lacking interconnected reticular networks and the continual fission and fusion observed in many other cell types. Nevertheless, proteins essential to mitochondrial network remodeling are abundant in adult hearts. Recent findings from cardiac-specific ablation of mitochondrial fission and fusion protein genes have revealed unexpected roles for mitochondrial dynamics factors in mitophagic mitochondrial quality control. This overview examines the clinical and experimental evidence for and against a meaningful role for the mitochondrial dynamism–quality control interactome in normal and diseased hearts. Newly discovered functions of mitochondrial dynamics factors in maintaining optimal cardiac mitochondrial fitness suggest that deep interrogation of clinical cardiomyopathy is likely to reveal genetic variants that cause or modify cardiac disease through their effects on mitochondrial fission, fusion, and mitophagy.

## Introduction

**Figure 1:**
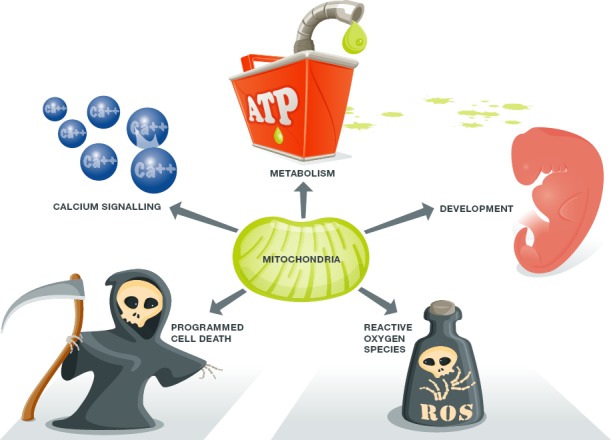
Schematic depiction of the extensive and varied impact of mitochondria on cell processes Mechanistic details of how mitochondria produce ATP and ROS are shown in Fig2.

The proposition that mitochondria are essential to many different aspects of normal heart functioning seems unassailable (Fig1). Mitochondria are the source of ATP that fuels excitation–contraction coupling. They sense smooth endoplasmic reticulum (ER) calcium release to adjust metabolism and anticipate actual physical need (Chen *et al*, 2012b; Dorn & Maack, 2013; Eisner *et al*, 2013). They are a major source of reactive oxygen species that (depending upon biological context and chemical concentration) can act as either physiological signals or damaging cytotoxins (Eisner *et al*, 2013; Song *et al*, 2014). Mitochondria are also the ‘gatekeepers’ of two forms of programmed cell death, apoptosis mediated by Bcl2 family mitochondrial death proteins and programmed necrosis mediated by opening of the mitochondrial permeability transition pore (Wei *et al*, 2001; Whelan *et al*, 2012; Dorn & Kitsis, 2015). Finally, mitochondria regulate cardiomyocyte differentiation and embryonic cardiac development (Kasahara *et al*, 2013; Cho *et al*, 2014). Given the multifaceted impact of mitochondria on cardiac development, minute by minute heart functioning, and cardiomyocyte suicide, one might predict that genetic mutations adversely impacting mitochondrial function would be a common cause of cardiac disease, as are mutations of sarcomeric proteins (Seidman & Seidman, 2011). This is not the case. Indeed, even severe primary mitochondrial diseases caused by loss-of-function nuclear or mitochondrial DNA mutations affecting mitochondrial electron transport chain complexes exhibit variable cardiac involvement (Holmgren *et al*, 2003). Furthermore, the heart is generally spared in human genetic diseases primarily impacting either the mitophagy mitochondrial quality control pathway (e.g., Parkinson's disease) or the mitochondrial dynamics apparatus (e.g., Charcot–Marie–Tooth syndrome and dominant optic atrophy) (Trinh & Farrer, 2013; Bombelli *et al*, 2014). Progressive neurological degeneration, and not heart disease, is the most common heritable feature in genetic disorders affecting mitophagy signaling and mitochondrial dynamism.

Given that hearts are the most mitochondria-rich organ, and indeed will only contract for a handful of cycles after interruption of mitochondrial ATP production, it seems paradoxical that cardiac disease is not more commonly manifested in primary mitochondrial disorders. There are several possible explanations: First, gene–gene and gene–environment interactions may tend to spare the heart. Diseases of primary mitochondrial dysfunction, such as Barth syndrome or Parkinson's disease were originally described as Mendelian or monogenic diseases. This appears to be an over-simplification, as phenotypic variation within a given kindred suggests that polygenic interactions and epigenetic factors can have a meaningful impact as disease modifiers. Likewise, the effect of environmental factors on mitochondrial disease can be substantial, as shown by Parkinson's disease that develops in some individuals exposed to pesticides (Goldman, 2014). Second, biological redundancy within affected mitochondrial pathways may protect the heart, allowing for compensatory induction of secondary metabolic, dynamism, or quality control pathways in the context of a single genetic ‘hit’. A third, generally overlooked, possibility is that hearts actually exhibit normal (or even enhanced) sensitivity to mitochondrial dysfunction. Because overt heart disease is rare in mitochondrial disorders, and neurological disease is more common, it is widely concluded that the neurological system is unusually sensitive, and the heart is relatively resistant, to primary mitochondrial dysfunction. A contrary interpretation is that for reasons of high cardiomyocyte mitochondrial density and unavoidable reliance of the heart upon constant mitochondria-supplied energy to sustain life, hearts are actually more sensitive and the reason that cardiac involvement is not commonly observed in disorders caused by severe underlying mitochondrial dysfunction is non-viability of affected individuals and evolutionary suppression in affected populations. In this context, the well-known metabolic plasticity and robustness of normal myocardium might be better able (than neurological tissue for example) to compensate for mitochondrial dysfunction in disorders caused by modest mitochondrial dysfunction, and thus severe cardiac involvement is not observed in the slowly progressive mitochondrial disorders that tend to attack the neurological system. In support of this notion, Parkinson's disease that affects neurological, but not cardiac, function impacts mitochondria only indirectly (i.e., by impairing their targeted elimination), and secondary mitochondrial quality control pathways have been identified that appear to spare cardiomyocytes from the type of cellular degeneration typically restricted to dopaminergic neurons.

In the following review, I revisit standard notions of mitochondrial dynamism and quality control in light of recent data elucidating important roles for mitochondrial fission and fusion in maintaining cardiac mitochondrial fitness. Based on emerging data, I make the case that fresh efforts employing next-generation DNA sequencing to discover and define genetic mitochondrial defects in unexplained cardiomyopathy (Man *et al*, 2013) are likely to uncover novel rare and pathophysiologically significant primary mitochondrial disorders of the heart.

## Overview of mitochondrial dynamism and mitophagy

Mitochondria are capable of modulating their shape and inter-organelle connectivity by fusion or fission events. The molecular mechanisms by which such events occur have been thoroughly described (Chan, 2012) and are not reproduced in detail here. It is notable, however, that cardiomyocyte mitochondria of fully developed normal hearts are ovoid in shape and not connected into the networks observed in other cell types. Cardiomyocyte mitochondria do not normally vary greatly in size, either as individual organelles within the greater cell population or over time. Fission and fusion of these organelles are sufficiently uncommon in hearts as to be nearly impossible to observe (Dorn & Kitsis, 2015; Song & Dorn, 2015). Thus, the heart is an exception to the often stated generalization that mitochondria are ‘highly dynamic’ (Otera & Mihara, 2011). Nevertheless, there is compelling inferential evidence that cardiomyocyte mitochondria of adult mice exhibit some degree of structural dynamism: Preventing their fusion causes mitochondria to become smaller (from unopposed fission), and preventing their fission produces mitochondrial enlargement (from unopposed fusion) (Song *et al*, 2015). Importantly, cardiomyocyte-specific genetic interruption of either mitochondrial fusion or fission is incompatible with mammalian life (Chen *et al*, 2011; Papanicolaou *et al*, 2012; Kasahara *et al*, 2013; Kageyama *et al*, 2014; Ishihara *et al*, 2015; Song *et al*, 2015). The requirement for cardiac mitochondrial fission and fusion factors in the absence of readily observable cardiomyocyte mitochondrial dynamism suggests involvement of mitochondrial fission and fusion proteins in other essential mitochondrial functions. To provide background information prior to examining possible alternate functioning of mitochondrial fission and fusion factors in hearts, I first briefly review cardiac involvement in prototypical mitochondrial diseases.

## Cardiac involvement in primary genetic mitochondrial respiratory dysfunction

Cardiac involvement in primary mitochondrial disease is surprisingly uncommon and limited to a few hallmark conditions. The prototypical genetic mitochondrial disease affecting the heart is Barth syndrome, caused by mutations in the *TAZ* gene (Barth *et al*, 1983). The encoded protein, tafazzin, is a mitochondrial inner membrane phospholipid transacylase involved in cardiolipin maturation (Vreken *et al*, 2000). This X-linked disease is extremely rare (∽1/300,000 births) and is characterized by neutropenia, cardiac and skeletal myopathies, growth delay, and elevated urine levels of 3-methylglutaconic acid (Cantlay *et al*, 1999). Cardiac involvement in the form of dilated or hypertrophic cardiomyopathy, with or without arrhythmias, is seen in over 90% of subjects (Roberts *et al*, 2012). Ventricular non-compaction/hyper-trabeculation has been described (Towbin, 2010). Mitochondria in Barth syndrome patients show hyper-proliferation and partial dissipation of the inner membrane potential (Xu *et al*, 2005). In a *Drosophila* Barth syndrome model, indirect flight muscle mitochondria exhibited abnormal cristae (Xu *et al*, 2006) similar to the stacks of electron-dense cristae described in soleus muscles of a *TAZ* shRNA mouse model (Soustek *et al*, 2011); cardiac mitochondria are greatly enlarged. Mitochondrial proliferation, aggregation, and abnormal cristae are also observed in a *TAZ* shRNA mouse (Acehan *et al*, 2011). The specific mitochondrial mechanisms that cause cardiac and skeletal myopathies in Barth syndrome have not yet been described.

**Figure 2:**
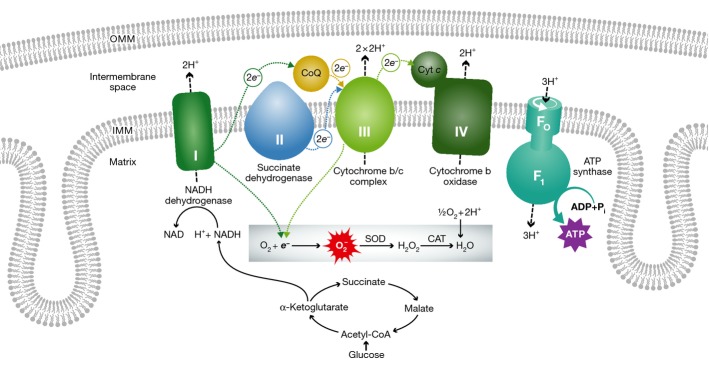
The mitochondrial electron transport chain (ETC.) and its relationship to ROS production Each of the inner mitochondrial membrane (IMM) ETC. enzymatic complexes is colored and indicated by their respective roman numerals (I-V). Electrons are transferred between complexes (dotted arrows), promoting proton (H^+^) transport (dashed arrows) from the matrix to the intermembrane space. Proton flow through ATP synthase (complex V) converts ADP to ATP. Normally, O_2_ is the terminal electron (e^−^) acceptor from complex IV. Electron leak from complexes I or III of damaged mitochondria can produce toxic reactive 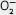 and H_2_O_2_. Friedreich's ataxia is caused by mutations in frataxin that induces dysfunction in complexes I, II, and III. Mitochondrial DNA mutations can affect complexes I, III, IV, and V, but not complex II that is comprised entirely of nuclear-encoded proteins.

A second example of heart disease provoked by primary genetic mitochondrial dysfunction is Friedreich's ataxia. This autosomal recessive neurodegenerative disease is caused by triplet nucleotide repeat expansions within the *FXN* gene, encoding the mitochondrial matrix iron chaperone protein frataxin (Campuzano *et al*, 1996; Durr *et al*, 1996). Loss of frataxin leads to dysfunction of iron cluster proteins in mitochondrial electron transport chain complexes I, II, and III (Rotig *et al*, 1997) (Fig2). In addition to progressive dorsal sensory nerve degeneration and ataxia, hypertrophic cardiomyopathy is seen in a majority of patients; heart failure is the terminal diagnosis in approximately one-third of affected individuals (Kipps *et al*, 2009; Tsou *et al*, 2011). Cardiac mitochondria in Friedreich's ataxia undergo massive proliferation with decreased ATP production (Lodi *et al*, 2001; Bunse *et al*, 2003), consistent with functional compromise of the electron transport chain. Primary mitochondrial defects and cardiomyopathy of Friedreich's ataxia are faithfully recapitulated in mouse models (Puccio *et al*, 2001; Whitnall *et al*, 2008).

Cardiac abnormalities are increasingly being discovered in disorders caused by mutations in mitochondrial DNA (mtDNA). The human mitochondrial genome is a ∽16,500-bp circular double-stranded mini-chromosome on which are encoded 13 proteins of the electron transport system (Fig2), 2 ribosomal RNAs, and 22 transfer RNAs. Mammalian mitochondria are passed through the maternal lineage, and therefore, mutations in mtDNA are maternally inherited. It is estimated that 1/5,000 individuals have some form of mtDNA mutational disease, approximately 50% of which will exhibit atypical hypertrophic cardiomyopathy with or without conduction disease (Elliott *et al*, 2008; Limongelli *et al*, 2010).

Mitochondrial proliferation and functional impairment are common features underlying cardiac disease in Barth syndrome, Friedreich's ataxia, and mtDNA mutation disease. It has been suggested that increased mitochondrial mass contributes to cardiomyocyte dysfunction by creating an internal resistance element (Wredenberg *et al*, 2002; Russell *et al*, 2004; Sebastiani *et al*, 2007). Progressive cardiac hypertrophy (Limongelli *et al*, 2010) in these primary mitochondrial diseases might be therefore explained as follows: Primary mitochondrial respiratory impairment can initially be compensated for, but over time is aggravated by secondary stochastic mitochondrial damage. The double hit of intrinsic mitochondrial dysfunction and age-related impairment provokes compensatory mitochondrial biogenesis, manifested as mitochondrial proliferation. Abnormal accumulation of interfibrillar mitochondria increases internal resistance to cardiomyocyte shortening, thereby stimulating cardiac hypertrophy. This mechanism, while consistent with the available data, remains speculative at this time. Genetically manipulated mouse models might be used to test the hypothesis that mitochondrial proliferation, in the absence of respiratory dysfunction, can physically impede unloaded cardiomyocyte shortening.

## Absence of cardiac phenotypes in primary disorders of mitochondrial quality control

**Figure 3:**
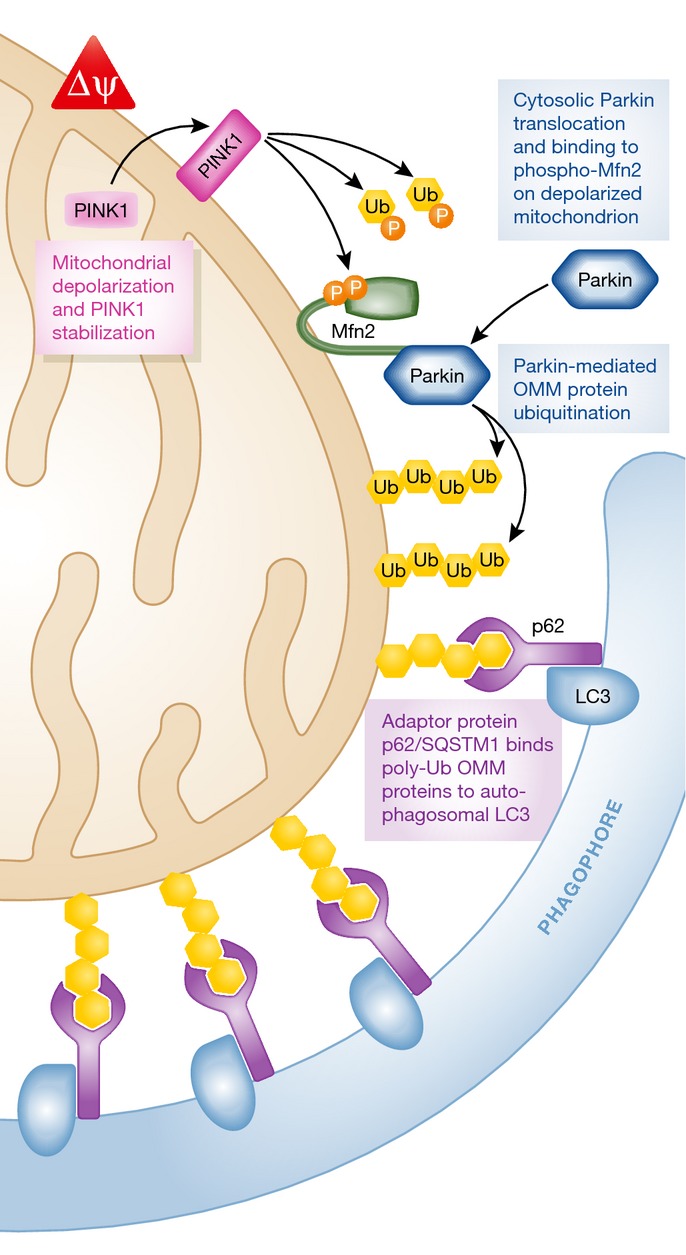
Molecular crosstalk between mitochondrial fusion and Parkin-mediated mitophagy A damaged/depolarized mitochondrion is shown in which PINK1 kinase levels have stabilized, resulting in phosphorylation of Mfn2 and its recruitment of Parkin. Poly-ubiquitination of outer mitochondrial membrane proteins by Parkin initiates mitophagy, ultimately resulting in lysosomal degradation.

The archetypal human disorder linked to a primary defect in mitochondrial fitness is early-onset autosomal recessive Parkinson's disease caused by loss-of-function mutations of either the *PARK2* (encoding Parkin) or *PARK6* (encoding PINK1) genes (Lees *et al*, 2009). Parkin and PINK1 are central effectors for targeted mitophagic elimination of dysfunctional mitochondria (Youle & Narendra, 2011). As illustrated in Fig3, PINK1 kinase is selectively stabilized in mitochondria that have diminished electrochemical potential across their inner membrane, recruits Parkin to mitochondria, and activates its E3 ubiquitin ligase activity. Parkin-mediated ubiquitination of mitochondrial outer membrane proteins is the signal for organelle engulfment by autophagosomes and subsequent transfer to degradative lysosomes, from whence the components are either recycled or disposed. Interruption of PINK1/Parkin-mediated mitophagy, as by loss-of-function Parkinson's disease mutations, suppresses a major mechanism for maintaining the overall quality of mitochondrial health: selective elimination of dysfunctional mitochondria. Epidemiologically, mutations of the *Parkin* gene are the major cause of early-onset hereditary Parkinson's disease; *PINK1* gene mutations are a less common cause (Lees *et al*, 2009). Mutations of either mitophagy factor induce degeneration and loss of dopaminergic neurons of the substantia nigra and locus coeruleus (Gesi *et al*, 2000). The underlying reasons for why dopaminergic neurons are particularly susceptible to cell damage from damaged mitochondria that were not properly eliminated, and therefore for the primarily neurological aspects of the disease, are poorly understood. Whereas mitophagy-defective mitochondria in iPSc-derived neurons from these patients appear overly abundant and dysmorphic (Seibler *et al*, 2011; Imaizumi *et al*, 2012; Rakovic *et al*, 2013), similar abnormalities are observed in cultured fibroblasts and skeletal muscle biopsies of patients with *Parkin* gene mutations (Ahlqvist *et al*, 1975; van der Merwe *et al*, 2014). Despite evidence for multi-system involvement, cardiac involvement in Parkinson's disease has only been inferred from statistical associations (Zesiewicz *et al*, 2004; Perez-Lloret *et al*, 2014).

The prototypical human diseases caused by interruption of mitochondrial fusion are Charcot–Marie–Tooth syndrome type 2A (CMT2A) and autosomal dominant optic atrophy (DOA). These heritable disorders have been linked to loss-of-function mutations in the mitochondrial fusion proteins, Mfn2 and Opa1, respectively (Burte *et al*, 2015). Mfn2 is one of two outer mitochondrial membrane GTPases that mediate mitochondrial tethering and outer membrane fusion, the first two events in organelle fusion (Chen & Chan, 2005). Additionally, Mfn2 uniquely bridges mitochondria to endo/sarcoplasmic reticulum, thus facilitating inter-organelle calcium transport and signaling (de Brito & Scorrano, 2010; Dorn & Maack, 2013; Dorn *et al*, 2015). Finally, PINK1-phosphorylated Mfn2 can act as the mitochondrial receptor for Parkin, giving it a role in mitophagy signaling (Chen & Dorn, 2013; Song *et al*, 2014) (Fig3). Because of this multi-functionality, *MFN2* mutations have the potential to adversely impact mitochondrial structure by interrupting organelle fusion, calcium uptake by interrupting ER/SR mitochondrial crosstalk, or mitophagy signaling by interrupting Parkin signaling (Fig3).

As with Barth syndrome and Friedreich's ataxia, mitochondrial proliferation is frequently described in CMT2A (Renaldo *et al*, 2012); the mitochondria themselves may be either reduced in size (Renaldo *et al*, 2012) or enlarged (Rouzier *et al*, 2012). Although CMT2A is classically described as a degenerative neurological disease of peripheral motor neurons with variable sensory nerve involvement, there can be substantial phenotypic overlap between CMT2A (typically caused by mutations of *MFN2*) and DOA (typically caused by mutations of *OPA1*); retinal involvement is described in 12% of CMT2A patients with confirmed *MFN2* mutations (Bombelli *et al*, 2014). Histological studies of skeletal muscle showing impaired mitochondrial functional integrity (using a stain for cytochrome oxidase activity) suggest that muscle involvement can be extensive (Rouzier *et al*, 2012). However, the question of whether myocardium is similarly affected in CMT2A caused by *MFN2* mutations remains unanswered.

As introduced, DOA is a disease caused by truncation mutations of the mitochondrial inner membrane fusion protein, Opa1 (Chen & Chan, 2010; Yu-Wai-Man & Chinnery, 2013). In addition to promoting IMM fusion, Opa1 plays a critical role for maintaining cristae structure and thereby in maintaining functional integrity of respiratory complexes located on the IMM (Pellegrini & Scorrano, 2007; Cogliati *et al*, 2013). Conventionally, DOA is characterized by loss of inner retinal ganglion cells, degeneration of the optic nerve, and progressive central loss of vision. Patients with a variant disorder called ‘DOA plus’ exhibit multi-system involvement with myopathy and peripheral neuropathy (Amati-Bonneau *et al*, 2008). This again demonstrates phenotypic overlap between CMT2A (caused by mutations of the outer mitochondrial membrane fusion protein Mfn2) and DOA (caused by mutations of the inner mitochondrial fusion protein Opa1). Manipulation of the homologous *Drosophila* genes has been useful in uncovering differences in the cell pathology induced by outer vs. inner mitochondrial membrane fusion defects. Thus, suppressing reactive oxygen species (ROS) markedly improves eye and heart tube phenotypes induced by RNAi-mediated Opa1 suppression, but not by mitofusin suppression (Yarosh *et al*, 2008; Bhandari *et al*, 2014b). By contrast, skeletal and cardiac myopathies induced by mitofusin deficiency in *Drosophila* are improved by enhancing the ability to handle ER stress (Bhandari *et al*, 2014b; Debattisti *et al*, 2014), which was ineffective in Opa1 insufficiency.

## Insights from genetic mouse models

The virtual absence of clinical cardiac disease in genetic disorders of mitochondrial quality control (Parkinson's disease) and mitochondrial dynamism (Charcot–Marie–Tooth syndrome) seems inconsistent with cardiac involvement in mitochondrial diseases such as Barth syndrome and Friedreich's ataxia that are linked to respiratory chain dysfunction. In trying to understand the reasons for these differences, it is worth noting that the seemingly straightforward functional subclassification of PINK1 and Parkin mutations as principally affecting mitochondrial quality control, and Mfn2 and Opa1 mutations as primarily impacting mitochondrial dynamism, may be misleading. In addition to mitophagy, PINK1/Parkin signaling can provoke mitochondrial fragmentation (Deng *et al*, 2008; Poole *et al*, 2010; Yu *et al*, 2011; Rana *et al*, 2013; Buhlman *et al*, 2014; Sugiura *et al*, 2014). Likewise, PINK1-phosphorylated Mfn2 helps recruit Parkin to damaged mitochondria during mitophagy (Chen & Dorn, 2013; Chen *et al*, 2014), and mitochondrial fission induced by Drp1 appears to be essential for normal mitochondrial quality control (Song *et al*, 2015). Thus, functional overlap between pathways allows for different interpretations of genetic ablation studies.

**Figure 4:**
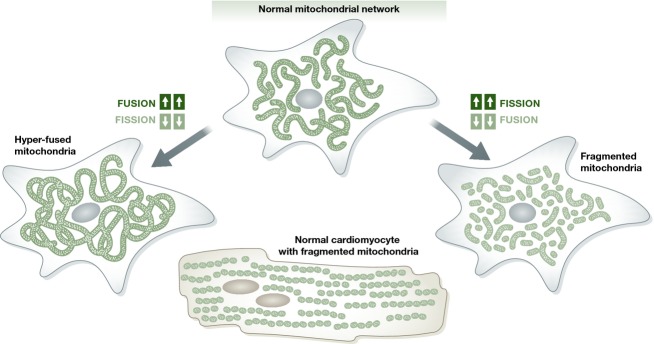
Mitochondrial structure and the role of mitochondrial dynamism in fibroblasts and cardiomyocytes A prototypical fibroblast-like cell is shown at the top with interconnected filamentous mitochondria (green). Mitochondrial hyper-connectivity occurs if the balance between fission and fusion is shifted toward fusion (left), whereas fragmentation occurs if the balance is shifted toward fission (right). Normal adult cardiomyocytes (bottom) have intrinsically ‘fragmented’ mitochondria and lack observable organelle remodeling.

Before examining the phenotypes obtained after tissue-specific ablation of mitochondrial fusion or fission factors, it may be useful to review lessons learned from cell biology that preceded the *in vivo* studies. Unlike the ovoid individual mitochondria of cardiomyocytes, many cells have interconnected filamentous mitochondrial networks. These networks undergo structural remodeling via fission-mediated disassembly and fusion-mediated re-assembly. When fission and fusion are at equilibrium, overall mitochondrial size is stable (Fig4). Fibroblasts typically have elongated organelles with a length/width aspect ratio of ∽6 (Song *et al*, 2015). Increasing mitochondrial fusion and/or inhibiting organelle fission will result in elongated mitochondria and increased network interconnectivity. Under these conditions, mitochondrial networks may even appear continuous throughout the cell (Song *et al*, 2015). Conversely, inhibiting mitochondrial fusion and/or increasing the rate of organelle fission results in numerous unconnected shorter organelles, commonly referred to as ‘fragmented mitochondria’ (Chen *et al*, 2003; Ishihara *et al*, 2009; Song *et al*, 2009, 2015). It is generally accepted that increased mitochondrial connectivity produced by shifting the balance of mitochondrial dynamism toward either more fusion or less fission represents a more physiological condition, and that less connectivity or fragmentation provoked by increased fission or impaired fusion is pathological. In other words, mitochondrial fusion is ‘good’, but mitochondrial fission is ‘bad’. The notion that fragmented mitochondria are detrimental probably derives from early observations that mitochondria networks undergo fission-mediated dissolution during apoptosis, linking the process of mitochondrial fission to programmed cell death (Suen *et al*, 2008). Consistent with this idea, inhibition of Drp1-induced mitochondrial fission using mdivi-1 or other agents can protect against cardiac or brain injury after ischemic damage (Lackner & Nunnari, 2010; Ong *et al*, 2010; Disatnik *et al*, 2013; Guo *et al*, 2013; Sharp *et al*, 2014). Furthermore, fragmented mitochondria of mitofusin-deficient MEFs appear partially depolarized, reflecting compromised respiratory function, whereas mitochondria of Mfn1 or Mfn2 single null MEFs appear fully polarized and only modestly shorter than normal (Chen *et al*, 2003; Song *et al*, 2007, 2015). Thus, there appears to be a direct relationship between mitochondrial fusion and mitochondrial health. Indeed, as Mfn1 and Mfn2 can interact both homo- and hetero-typically, overexpressing Mfn1 largely corrects abnormalities caused by the absence of Mfn2, and vice versa, and the modest disturbance in mitochondrial morphometry in single Mfn1 or Mfn2 knockout MEFs (Chen *et al*, 2003) is likely the consequence of an insufficiency in total mitofusin protein (analogous to haploinsufficiency of two functionally synonymous genes).

Accumulating evidence suggests that the proposition that mitochondrial fusion is ‘good’ and fission is ‘bad’ is an over-simplification and that cell death linked to mitochondrial fission is contextual: First, adult cardiomyocyte mitochondria are intrinsically fragmented in comparison with other cells, that is, they are typically small (aspect ratio ∽1.5) and not connected into networks (Fig4). Nevertheless, hearts function well and cardiomyocyte apoptosis is not normally observed. Second, highly fused mitochondria exhibit greater sensitivity to staurosporine-stimulated apoptosis, not protection as would be predicted based on conventional wisdom (Szabadkai *et al*, 2004). Third, genetic deficiency of the mitochondrial fusion factor Mfn2 actually protects against cell necrosis (Whelan *et al*, 2012). And finally, spontaneous mitochondrial fragmentation induced by Drp1 overexpression fails to provoke meaningful pathology, at least in *Drosophila* cardiomyocytes (Bhandari *et al*, 2014b). Changes in mitochondrial shape and connectivity after genetic mitofusin ablation *in vitro* (Chen *et al*, 2005) or *in vivo* (Chen *et al*, 2007, 2010; Pham *et al*, 2012) may not therefore be the only (or actual) cause of observed cellular dysfunction and disease phenotypes.

Examining the consequences of interrupting mitochondrial fusion raises further considerations. Mitochondrial fusion is the opposite of fission. Thus, if it is correct that fission is detrimental, then it is common sense that fusion should be beneficial. For example, a fusion event between one damaged and one undamaged mitochondrion has the potential to dilute out damaged components in the former, thereby repairing the damaged organelle via the process of ‘functional complementation’ (Youle & van der Bliek, 2012). However, this schema ignores the potential for bidirectional exchange of components. Accordingly, a fusion event between a damaged and healthy organelle would not only dilute out the damaged components in the former, but contaminate the healthy components of the latter; fusion has the potential to harm the originally healthy organelle to the same extent that it benefits the impaired one. Indeed, our laboratory recently demonstrated fusion-mediated contamination of good mitochondria by bad, which we refer to as ‘mitochondrial contagion’. In these studies, heart tube dysfunction and loss of mitochondrial fitness in Parkin-deficient *Drosophila* cardiomyocytes were delayed by inhibiting mitochondrial fusion through RNA-mediated suppression of the *Drosophila* mitofusin, MARF (Bhandari *et al*, 2014a). Thus, in this instance, mitochondrial fusion was detrimental and its inhibition was therapeutic. Accumulating data indicate that mitochondrial fusion and fission are neither inherently good or bad, but that a proper balance between the two is important both in cells with interconnected mitochondrial networks and in cardiomyocytes.

### *In vivo* mitofusin deletion

Deleting mitofusin genes that encode essential OMM mitochondrial fusion proteins was predicted to impair mitochondrial fusion. However, because Mfn1 and Mfn2 can mutually substitute for one another as mitochondrial tethers and OMM fusion proteins (Chen *et al*, 2003), only their combined ablation abrogates Mfn-mediated mitochondrial fusion. Nevertheless, germ line ablation of either Mfn1 or Mfn2 genes is embryonic lethal, likely because the absence of either mitofusin adversely impacts placental development (Chen *et al*, 2003). For this reason, the expanding body of knowledge regarding consequences of *in vivo* loss of mitofusins derives from conditional gene ablation using the floxed allele mice generated by David Chan and colleagues (Chen *et al*, 2007). In general, combined Mfn1/Mfn2 ablation in neurons (Chen *et al*, 2007), skeletal muscle (Chen *et al*, 2010), and myocardium (Chen *et al*, 2011), which should provoke total loss of mitochondrial fusion in the affected cell types (Chen & Chan, 2010), produced severe abnormalities, whereas individual deletion of either Mfn1 or Mfn2 in the same tissues produced comparatively minor effects in the same tissues (Chen *et al*, 2007, 2010, 2012b) (Chen & Dorn, 2013). For example, combined ablation of Mfn1 and Mfn2 in skeletal muscle induced skeletal myopathy with mitochondrial fragmentation, increased mtDNA mutations, loss of mtDNA, and mitochondrial proliferation (Chen *et al*, 2010). From this constellation of findings, it was concluded that loss of mitochondrial fusion causes instability in mitochondrial DNA and provokes compensatory mitochondrial proliferation. However, recent findings that deletion of Mfn2 (either alone or in combination with Mfn1) can directly impair mitochondrial quality control suggest an additional contributory mechanism.

Notwithstanding major functional redundancies between Mfn1 and Mfn2 as promoters of mitochondrial fusion in MEFs, there are intriguing differences when the two mitofusins are individually deleted in mouse tissues using Cre-lox strategies. In dopaminergic neurons, Slc6a3-Cre-mediated deletion of floxed Mfn1 was well tolerated, whereas deletion of Mfn2 provoked mitochondrial abnormalities, neuronal death, and a Parkinson's disease-like neuromuscular phenotype (Pham *et al*, 2012). Likewise, ablating mitofusins in the dopaminergic neurons of floxed allele mice using a dopamine amino transferase (DAT)-Cre transgene (Lee *et al*, 2012) revealed that Mfn1 deletion is well tolerated, whereas loss of Mfn2 evoked severe locomotor defects and early lethality. Intriguingly, mitochondria of Mfn2-deficient neurons were markedly enlarged, and not fragmented as would be anticipated if Mfn2 deletion had prompted a mitochondrial fusion defect. Importantly, Mfn2-deficient neuronal mitochondria specifically exhibited a defect in Parkin binding (Lee *et al*, 2012).

The results obtained after Mfn1 or Mfn2 ablation in the brain resemble those of cardiac-specific Mfn1 and Mfn2 deletion. Cardiomyocyte Mfn1 deficiency is well tolerated, but cardiomyocyte Mfn2 deficiency causes mitochondrial enlargement (Papanicolaou *et al*, 2011; Chen *et al*, 2012b; Chen & Dorn, 2013) with increased mitochondrial ROS production (Song *et al*, 2014) and an interruption of normal cytosolic–mitochondrial Parkin translocation (Chen & Dorn, 2013). A common theme from these studies is that loss of Mfn2 induces more severe phenotypes than loss of Mfn1, suggesting some action of Mfn2 in addition to its titular role in promoting mitochondrial fusion. Indeed, since interfering with mitochondrial fusion produces mitochondrial shortening both *in vitro* and *in vivo* (Chen *et al*, 2003, 2011; Song *et al*, 2015), mitochondrial enlargement in multiple models of organ-specific *in vivo* Mfn2 insufficiency seems to rule out defective fusion as the principal causal mechanism.

### *In vivo* Drp1 deletion

If the conventional notion is correct that smaller fragmented mitochondria are ‘bad’ and that larger more interconnected mitochondria are ‘good’, then genetic interruption of mitochondrial fission by ablating Drp1 should be beneficial (as was the case with pharmacological Drp1 inhibition in cardiac ischemic injury (Disatnik *et al*, 2013; Ong *et al*, 2010)). However, the results of four recent studies that examined the consequences of ablating Drp1 in mouse hearts provide additional evidence that larger mitochondria are not necessarily better mitochondria. Three groups independently deleted Drp1 in cardiomyocytes by combining Drp1 floxed allele mice with either constitutive or tamoxifen-inducible α-myosin heavy chain Cre transgenes (Kageyama *et al*, 2014; Ikeda *et al*, 2015; Song *et al*, 2015). As expected, Drp1-deficient cardiomyocyte mitochondria were enlarged (from unopposed mitochondrial fusion). Unexpectedly, cardiomyopathies developed in each study and were linked in different ways to disturbances of mitochondrial autophagy. In a fourth study, neonatal cardiomyopathy developed in mice with a more generalized muscle-specific deletion of Drp1 (Ishihara *et al*, 2015). Thus, cardiomyocyte-specific *in vivo* inhibition of mitochondrial fission has revealed another level of crosstalk between mitochondrial dynamism and quality control.

**Figure 5:**
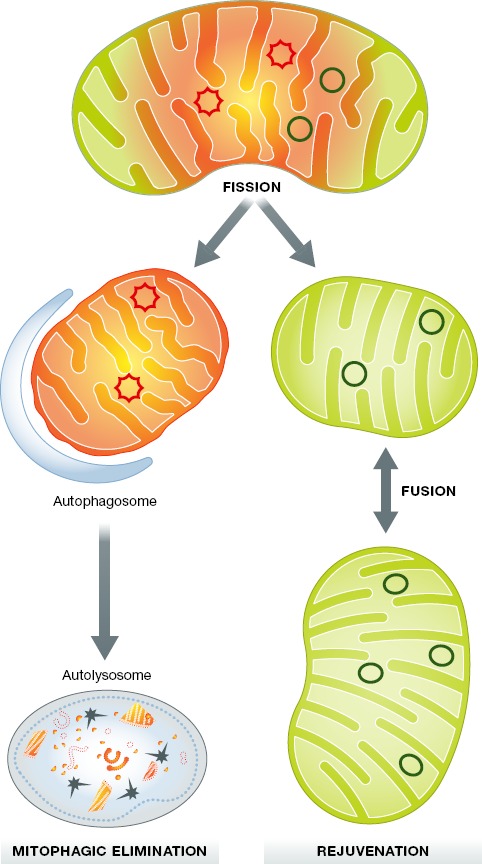
Asymmetric fission for mitochondrial quality control A damaged or senescent mitochondrion (mixture of damaged yellow/orange and healthy green components) is shown. Via asymmetric fission, the healthy components are delivered to one daughter that is fusion-competent, rejuvenated, and retained. The damaged components are segregated into the other, smaller daughter that is depolarized and promptly eliminated via mitophagy.

The most straightforward explanation for how defective mitochondrial fusion or fission can adversely impact mitophagy is that an intact fission–fusion cycle is essential for asymmetric mitochondrial fission in which damaged or malfunctioning mitochondrial components are segregated from healthy components and targeted for removal (Twig *et al*, 2008; Dorn, 2015) (Fig5). However, a fission–fusion cycle defect does not explain why Mfn2 ablation produces greater mitochondrial dysfunction than Mfn1 ablation (see above), or reveal why Parkin translocation defects in neurons and cardiomyocytes are exclusively evoked by Mfn2 deficiency (Lee *et al*, 2012; Pham *et al*, 2012; Chen & Dorn, 2013). A molecular mechanism was provided by our observation that Mfn2 is enabled as a mitochondrial-localized binding protein for Parkin after its phosphorylation by the PINK1 kinase (Chen & Dorn, 2013). PINK1 is preferentially stabilized in damaged/depolarized mitochondria, with the consequence that Parkin translocates to that organelle and stimulates its mitophagy by ubiquitinating mitochondrial proteins (see Fig3; Narendra *et al*, 2010). In our *in vivo* studies, Mfn2 is an essential intermediate in the interaction between mitochondrial PINK1 and cytosolic Parkin, being phosphorylated by the former and thereby transformed into a mitochondrial binding partner for the latter (Chen & Dorn, 2013). The dual role of Mfn2 as both mitochondrial fusion factor and Parkin receptor in the mitophagy appears sufficient to explain mitochondrial enlargement, degeneration, and proliferation in Mfn2 deficiency in the heart (Dorn & Kitsis, 2015) and brain (Lee *et al*, 2012).

### *In vivo* Opa1 deletion

Mitofusins and Drp1 mediate the initial steps of mitochondrial fusion and fission, respectively. However, complete mitochondrial integration after OMM fusion requires Opa1-mediated fusion of the IMM. Intriguingly, insufficiency of Opa1 produces cardiac phenotypes in *Drosophila* and mice that are distinct from those induced by mitofusin ablation. Perhaps these differences are attributable to important Opa1 functions in addition to mediating inner mitochondrial membrane fusion, such as being a critical modulator of cristae architecture (and therefore of respiratory complex functioning) (Frezza *et al*, 2006; Cogliati *et al*, 2013). Consistent with this reasoning, germ line Opa1 haploinsufficiency in mice induces mitochondrial fragmentation and ROS production with an age-dependent cardiomyopathy (Chen *et al*, 2012a). The importance of respiratory compromise and ROS production in heart disease provoked by Opa1 deficiency was demonstrated in *Drosophila* heart tubes wherein RNAi-mediated Opa1 suppression produced not only small mitochondrial dysmorphology and cardiomyopathy, but markedly increased cardiomyocyte ROS production. Suppressing cardiomyocyte mitochondrial ROS production with transgencially expressed superoxide dismutase improved the cardiomyopathy, whereas enhancing the ability of heart tubes to manage ER stress had no effect. This is the reciprocal approach to the one that improved the cardiomyopathy of *Drosophila* mitofusin deficiency in the same study; ROS inhibition was ineffective, whereas improving the ER stress response rescued heart tube dysfunction (Bhandari *et al*, 2014b). Thus, unlike deficiency of OMM mitofusin proteins that impairs culling of dysfunctional mitochondria exhibiting age-related or extrinsic damage, Opa1 deficiency induces primary mitochondrial respiratory dysfunction, in part by producing poorly tolerated abnormalities of cristae respiratory supercomplexes.

### Mouse models of primary mitophagic dysfunction

Functional crosstalk between mitochondrial dynamics and mitophagy pathways complicates interpretation of cardiac gene manipulation studies. The following questions arise: Are resulting phenotypes the direct consequence of altering the balance between mitochondrial fission and fusion? Are they an indirect effect that perturbing mitochondrial dynamism exerts on mitochondrial quality control signaling? Answers to these questions may be found in studies of mice lacking the two central effectors of mitophagy, PINK1, and Parkin.

Germ line deletion of the murine PINK1 gene provoked progressive cardiac dysfunction beginning at 2 months of age (Billia *et al*, 2011). Although apoptosis was reported as being increased, the overall cardiac phenotype was cardiac hypertrophy, and not dilated cardiomyopathy that typically results from increased cardiomyocyte death. Mitophagy was not measured, but diminished mitochondrial respiratory function and respiratory complex content and increased oxidative stress are consistent with decreased mitochondrial quality. Notably, mitochondrial size was increased. Thus, interruption of mitophagy by PINK1 deletion is detrimental to hearts.

Germ line ablation of the Parkin gene is perhaps most noteworthy for the absence of major phenotypes, cardiac, neurological, or otherwise. None of the multiple types of Parkin knockout mice develop a Parkinson's disease-like phenotype (Dawson *et al*, 2010), perhaps because of opportunistic compensation by related E3 ubiquitin ligases (Bhandari *et al*, 2014a) or other mitophagy factors (Shin *et al*, 2011). Nevertheless, modest cardiac and cardiomyocyte mitochondrial abnormalities were detected in 1-year-old Parkin null mice (Hoshino *et al*, 2013; Kubli *et al*, 2013). Taken together, these early findings in germ line PINK1 and Parkin knockout mice suggest that a common constellation of cardiac defects develops when mitophagy is impaired in cardiac myocytes. At the organelle level, there is accumulation of structurally abnormal, enlarged, dysfunctional mitochondria; at the organ level, there is a relatively modest and slowly progressive cardiomyopathy. These findings are similar to those provoked by cardiac Mfn2 deficiency that interrupts PINK1/Parkin signaling by deleting the mitochondrial Parkin receptor (Chen & Dorn, 2013; Song *et al*, 2014) and are striking in their contrast to the rapidly progressive and lethal cardiomyopathy induced by complete interruption of mitochondrial fusion (combined Mfn1 and Mfn2 gene ablation) (Chen *et al*, 2011; Papanicolaou *et al*, 2012). It seems reasonable to postulate that interruption of the mitochondrial fission–fusion cycle by combined ablation of both Mfn1 and Mfn2 (fusion-deficient), or deletion of Drp1 (fission-deficient), evokes severe and acutely deleterious effects on the heart that include, but are not limited to, interrupted mitochondrial quality control (Chen *et al*, 2011; Song *et al*, 2015). By comparison, deletion of Mfn2, PINK1, or Parkin evokes a chronic form of cardiac degeneration through loss of an important mechanism to maintain mitochondrial fitness, mitophagic culling. Cardiac degeneration produced by interrupting the PINK1/Mfn2/Parkin mitophagy pathway may be chronic and delayed because Parkin-independent pathways for mitochondrial quality control, such as the incompletely understood ROS-dependent mitophagy pathway (Song *et al*, 2014), are invoked but ultimately overwhelmed.

### Implications of experimental findings for clinical medicine

Barth syndrome, Friedreich's ataxia, and cardiac involvement in mtDNA disease are examples of primary mitochondrial dysfunction that adversely impact the heart. In each of these conditions, mitochondrial respiratory chain dysfunction is a central pathological feature. In other disorders such as Parkinson's disease, CMT2A, and DOA, the principal abnormality is not at the mitochondrion, but within the mitochondrial cell signaling interface that maintains fitness of the mitochondrial pool. Although mitochondrial respiratory dysfunction is measurable in cells derived from patients with these disorders and in the respective fly and mouse genetic models, the heart is seemingly spared from major involvement. Or perhaps not. A review of published literature reveals almost no direct information on cardiomyopathy in Parkinson's disease, CMT2A, or DOA (despite compelling evidence for skeletal muscle involvement in all three syndromes), and cardiac phenotypes have been described in mice lacking the causal genes for each of these neurodegenerative conditions. Given the rapid technical advances in deep sequencing of exomes and whole genomes, we may discover unsuspected mitochondrial involvement in hereditary cardiac and skeletal myopathies that are not explained by the usual common genetic disturbances in sarcomeric proteins, dystrophin, etc. Indeed, reports are beginning to surface of severe congenital cardiomyopathies attributed to primary abnormalities of mitochondrial respiratory chain function (Dhar *et al*, 2013; Garcia-Diaz *et al*, 2013).

Biology is complex, and complex things are by definition not simple. While this would appear to be stating the obvious, our tendency as researchers and clinicians can be to follow Occam's razor and default to the simplest explanation, assuming that it will be the best. Sometimes, however, simplicity just reflects a position low on the learning curve, and with additional knowledge a more complex and elegant conceptual paradigm is engendered. We are currently witnessing this type of evolutionary thinking about primary mitochondrial involvement in heart disease, and of cardiac involvement in primary mitochondrial disease. The conventional functional distinction between mitochondrial dynamism and quality control pathways is an example of how straightforward concepts are giving way to a more elegant understanding of integrated biological processes governing mitochondrial homeostasis. The concept of a mitochondrial dynamism–mitophagy interactome (Dorn & Kitsis, 2015) may be helpful in understanding phenotypic overlap and mechanistic uncertainties. Mfn2 is both a pro-fusion protein with effects similar to Mfn1 and an important factor in Parkin-mediated mitophagy. A mutation that impairs only the pro-fusion activity of Mfn2 might have little effect because residual Mfn1 can compensate. But if it acts as a dominant inhibitor of Mfn1- and Mfn2-mediated fusion, an *MFN2* mutation might completely interrupt outer membrane fusion, phenocopying combined Mfn1/Mfn2 ablation. Alternately, a mutation in Mfn2 that specifically affects Parkin binding might disrupt mitophagy while preserving mitochondrial fusion. The point is, in no instance is the effect of the human point mutation faithfully recapitulated by something as crude as *MFN2* gene ablation. For this reason, as rare mutations of mitochondrial dynamics and mitophagy factors are identified in disease cohorts, defining the causal disease mechanism will require a more complete understanding of the complex interplay between these (and other) pathways. This may best be accomplished for *in vitro* cell-based and *in vivo* murine experimental platforms using gene editing knockin, rather than knockout approaches.

**Pending issues**

1. What are the functionally important roles of mitochondrial fission and fusion factors in adult hearts?
2. Does Parkin-mediated mitophagy help to sustain cardiac mitochondrial fitness?
3. Will deep DNA sequencing uncover rare mitochondrial-associated cardiomyopathies?

## Abbreviations

Charcot–Marie–Tooth syndrome type 2A (CMT2A): Degenerative neurological condition caused by loss-of-function mutations of Mfn2 gene.
Dominant Optic Atrophy: Degenerative retinal disease caused by loss-of-function mutations of Opa1 gene.
Dynamin-related protein 1 (Drp1): GTPase that mediates mitochondrial fission by translocation, oligomerization, and constriction.
Inner mitochondrial membrane (IMM): The location of the electron transport complexes that mediated mitochondrial respiration; Opa1 maintains IMM structure.
Mitochondrial dynamism: Fission and fusion of mitochondria, typically viewed as a cyclic phenomenon that regulates mitochondrial shape and connectivity.
Mitofusins (Mfn): Two closely related mitochondrial proteins that mediate the initial steps in mitochondrial fusion: organelle tethering and outer membrane fusion.
Mitophagy: A contraction of mitochondria and autophagy that describes multiple mechanistically distinct processes in which cells identify and degrade dysfunctional mitochondria to maintain overall mitochondrial fitness.
Optic atrophy 1 (Opa1): Mitochondrial protein that mediates inner mitochondrial membrane fusion and is important for respiratory complex integrity. Mutations cause dominant optic atrophy.
Outer mitochondrial membrane (OMM): The membrane interface between mitochondria and parent cell; Parkin ubiquitinates OMM proteins, including Mfn1, Mfn2, and Drp1.
Parkin: The prototypical Parkinson's disease factor that, via its E3 ubiquitin ligase activity, plays a central role in PINK1/Parkin-mediated mitophagy.
Parkinson's disease: Chronic degenerative neuromuscular condition; early heritable forms are caused by loss-of-function mutations in genes encoding mitophagy factors Parkin or PINK1.
PTEN-induced putative kinase 1 (PINK1): Mitochondrial kinase that is preferentially stabilized in dysfunctional organelles to initiate mitophagy signaling via the Parkin pathway
